# Rose-derived extracellular vesicle-like particles enable anti-melanogenesis by suppressing the key melanogenic pathway in skin cells

**DOI:** 10.20517/evcna.2026.55

**Published:** 2026-09-04

**Authors:** Sirui Zhou, Huijing Li, Ying Yang, Weiying Zeng, Dongxiao Li, Tianxin Qiu, Weiguo Lu, Kewei Zhao

**Affiliations:** ^1^The Third Affiliated Hospital of Guangzhou University of Chinese Medicine, Guangdong Engineering Research Center of Chinese Herbal Vesicles, Guangzhou 510378, Guangdong, China.; ^2^The First Affiliated Hospital of Guangzhou University of Chinese Medicine, Guangzhou 510405, Guangdong, China.; ^3^The Third Clinical Medical College, Guangzhou University of Chinese Medicine, Guangzhou 510378, Guangdong, China.

**Keywords:** Rose-derived extracellular vesicle-like particles, melanogenesis, multidimensional molecular mechanism

## Abstract

**Aim:** Abnormal skin pigmentation is not merely a common cosmetic concern but is also associated with pigmentation disorders and an elevated risk of malignant lesions such as melanoma. To overcome the adverse effects of existing antimelanogenic agents, we developed rose-derived extracellular vesicle-like particles (RS-EVLP) as a novel and biocompatible melanogenesis inhibitor.

**Methods:** RS-EVLP were isolated from roses and characterized by transmission electron microscopy and mass spectrometry. Their anti-melanogenic effects were evaluated in B16 cells via melanin content and tyrosinase activity assays. Molecular mechanisms were investigated using molecular docking, molecular dynamics simulations, and surface plasmon resonance (SPR) analysis. *In vivo* efficacy was assessed in a zebrafish model, followed by a clinical trial in human volunteers.

**Results:** RS-EVLP exhibited a bilayer nanostructure (50-150 nm) and contained natural anti-melanogenic components, including luteolin and procyanidin B3. They showed high binding affinity to microphthalmia-associated transcription factor (MITF) and tyrosinase-related protein 1 (TRP-1). In B16 cells, RS-EVLP dose-dependently reduced melanin content and tyrosinase activity by downregulating MITF and the transcription of tyrosinase (TYR)/TRP-1/TRP-2 enzyme system. In zebrafish, RS-EVLP reduced melanin spot area by 24% ± 17.5%. Clinical studies confirmed that a 5% RS-EVLP formulation progressively enhanced skin brightness as skin penetration efficiency increased.

**Conclusion:** RS-EVLP overcomes the limitations in bioavailability and toxicity associated with traditional synthetic whitening agents, offering a promising natural alternative for developing whitening cosmeceuticals.

## INTRODUTION

The skin serves as a crucial defense barrier against external threats such as pathogens, ultraviolet radiation (UVR), and physical injury. By maintaining this barrier function, the body limits local and systemic consequences, including inflammatory responses, immune disorders, photoaging, metabolic abnormalities, and nutritional disorders, thereby supporting overall health^[1-3]^. The melanin system, composed of melanocytes and keratinocytes, plays a dual role in the skin^[4]^: regulating phenotypic pigmentation and absorbing UVR to provide photoprotection^[5,6]^. As the key mechanism of action, melanocytes deliver melanin-rich melanosomes through the cytosol to neighboring keratinocytes, forming protective supranuclear caps over the nuclei and effectively blocking UVR-induced DNA damage. Melanogenesis is precisely regulated by genetic, endocrine, and environmental factors^[7]^ via a molecular mechanism that begins with the oxidation of tyrosine to dopaquinone, followed by a series of enzymatic reactions leading to the maturation of melanosomes^[8,9]^. These pigment-laden organelles reach keratinocytes via dendritic transport to exert photoprotective effects^[10-12]^. The core pathway in this process is the alpha-melanocyte-stimulating hormone (α-MSH)/melanocortin 1 receptor (MC1R)-cyclic adenosine monophosphate (cAMP)-protein kinase A (PKA) signaling axis^[13,14]^. PKA-mediated phosphorylation of cAMP response element-binding protein (CREB) induces the expression of microphthalmia-associated transcription factor (MITF), a master regulator of melanogenic enzymes^[15-17]^. MITF is activated by binding its conserved M-box elements in the promoter region to coordinate the transcription of tyrosinase (TYR), tyrosinase-related protein 1 (TRP-1), and tyrosinase-related protein 2 (TRP-2)^[18-23]^. Interactions among cAMP, Wnt/β-linker proteins, and the mitogen-activated protein kinase/extracellular signal-regulated kinase (MAPK/ERK) pathway further fine-tune MITF activity, thereby forming a powerful pigment-controlled regulatory network.

Human skin contains melanin (both eumelanin and pheomelanin)^[24]^. The ratio of these two pigments determines skin color. An abnormal distribution of these pigments can lead to pigmentary disorders^[25]^, such as freckles and melasma, and is associated with malignant lesions, such as melanoma^[26,27]^. Modulating melanogenesis is key to treating pigmentary disorders. Current depigmenting agents (e.g., tretinoin, a TYR inhibitor, and arbutin, a hydroquinone derivative) reduce the amount of melanin, but can cause side effects, such as skin irritation, photosensitivity, and cytotoxicity^[28,29]^. Therefore, safer, plant-derived ingredients (e.g., licorice extract and soy isoflavones) have gained increasing attention^[30]^. However, plant components generally have low bioavailability and poor targeting, thus requiring novel delivery technologies to enhance efficacy^[31]^. Plant-derived extracellular vesicle-like particles (EVLPs) offer advantages in cellular uptake efficiency and biosafety. Notably, rose petal-derived extracellular vesicle-like nanovesicles (RPDNVs) have recently emerged as multifunctional tools for skin repair. Bae *et al*. demonstrated that RPDNVs, which are rich in antioxidants such as phenols and proteins, significantly promoted fibroblast migration and extracellular matrix remodeling without inducing a fibrotic response^[32]^. Moreover, they exhibited excellent skin tolerance and stability when applied in the relevant field. This indicates that rose-derived EVLP possesses an inherent biological activity associated with skin health. However, its potential to regulate melanin production has not been comprehensively explored. Recent studies of the mechanism employed by nanoparticles to regulate melanin have revealed that EVLPs derived from plant leaves and stems exert potent anti-melanogenic effects through UVR-dependent inhibition of the α-MSH-MC1R-MITF axis. This effect is superior to that of arbutin. Keratinocyte-derived exosomes containing miR-330-5p also inhibit melanogenesis^[32]^. Additionally, EVLPs of Korean *Codium fragile* and *Sargassum fusiforme* exhibit significant melanin inhibitory activity^[33]^.

This study systematically investigated the bioactivity and mechanism of action at the *in vitro*, *in vivo*, and clinical levels of rose-derived extracellular vesicle-like particles (RS-EVLP) to inhibit melanin activity. Electron microscopy revealed that RS-EVLP have a bilayer nanostructure ranging from 50 to 150 nm. Ultra-performance liquid chromatography coupled with quadrupole time-of-flight mass spectrometry confirmed the presence of the natural anti-melanogenic phytoconstituents luteolin and procyanidin B3. The combination of network pharmacology, molecular dynamics simulation, and surface plasmon resonance (SPR) technology found that the active components of RS-EVLP stably bind to MITF and TRP-1. Among these components, procyanidin B3, which is highly abundant in RS-EVLP, exhibited a significant binding affinity for TRP-1 [binding energy = -65.622 kJ/mol; root mean square deviation (RMSD) < 0.2 nm]. Mechanistic studies revealed that RS-EVLP inhibited melanin synthesis in B16 cells in a dose-dependent manner and significantly downregulated MITF expression and subsequent expression of the TYR/TRP-1/TRP-2 enzymatic system. A zebrafish model validated the *in vivo* whitening effect, showing a 24% ± 17.5% reduction in melanin spot area (*P* = 0.0103). Clinical trials demonstrated that a 5% RS-EVLP formulation significantly enhanced skin brightness and penetration efficiency in a time-dependent manner. These findings confirm the great potential of RS-EVLP for the development of skin-whitening products as a new therapeutic strategy for treatment of post-inflammatory hyperpigmentation, melasma, and other pigmentation disorders.

## METHODS

### Extraction and characterization of RS-EVLP

RS-EVLP were extracted from 500 g of dried *Rosa rugosa* flowers. First, the flowers were crushed using a pre-cooled grinder to obtain a crude extract, which was filtered. The filtrate was then centrifuged at 10,000 × *g* for 30 min. The resulting supernatant was filtered using a magnetic stirrer to remove molecules with molecular weights < 100 kD. The concentrated liquid was then centrifuged at 10,000 × *g* for 60 min and filtered through a 0.22-μm membrane (Tianjin Jinteng Experiment Equipment Co., Ltd., Tianjin, China) to remove large fragments. The RS-EVLP were concentrated by centrifugation at 100,000 × *g* for 70 min at 4 °C using an ultracentrifuge (Sorvall^TM^ WX+ Ultracentrifuge Series; Thermo Fisher Scientific, Waltham, MA, USA), resuspended in phosphate-buffered saline (PBS) (Wuhan Procell Life Science & Technology Co., Ltd., Wuhan, China), and stored at -80 °C. Prior to use, the RS-EVLP were concentrated by centrifugation at 100,000 × *g* for 70 min at 4 °C.

The particle size and concentration of the RS-EVLP were first determined using a nanoflow cytometer (NanoFCM Co., Ltd., Xiamen, China). To evaluate lipid membrane integrity, the RS-EVLP were subsequently treated with Triton X-100 for 30 min to rupture the vesicle membranes, after which the particle size and concentration were re-measured under the same conditions. The morphology of the RS-EVLP was observed with a transmission electron microscope (JEOL-1400; JEOL Ltd., Tokyo, Japan). In brief, each sample (10 μL) was placed on a copper mesh in a 6-cm Petri dish, allowed to settle for 10-30 min, then aspirated, stained with 10 μL of phosphotungstic acid for 30 s, and aspirated again. Afterward, the copper mesh was transferred to filter paper and shade-dried for at least 2 h prior to electron microscopy.

### Network pharmacology analysis and molecular docking

The active components of *R*. *rugosa* (i.e., flavonoids, phenolic acids, coumarins, and flavonol derivatives^[34-38]^) were analyzed by ultra-performance liquid chromatography coupled with quadrupole time-of-flight mass spectrometry, which revealed the presence of luteolin, ellagic acid, and proanthocyanidins. Based on the active ingredients reported in the literature and comparative analysis of rose hips by mass spectrometry, the key compounds of RS-EVLP were identified, and 47 core components were selected. Then, 372 potential targets were identified using the SwissTargetPrediction platform (http://www.swisstargetprediction.ch/). Based on the GeneCards database (https://www.genecards.org/) and the keyword “hyperpigmentation”, 1990 related genes were obtained. Finally, Cytoscape 3.9.1 bioinformatics software (https://cytoscape.org/release_notes_3_9_1.html) and the STRING database (https://string-db.org/) were used to construct multilevel interaction and protein-protein interaction networks. Additionally, Gene Ontology (GO) annotation pathway analysis of the common targets with reference to the Kyoto Encyclopedia of Genes and Genomes (KEGG) was performed using the Metascape platform (https://metascape.org/).

The mass spectra of the VLNPs of *Rosa moschata* and *Rosa sinensis* were compared, and the substances quercetin, luteolin, apigenin, kaempferol, ellagic acid, and procyanidin B3 were selected for molecular docking. After searching the RCSB Protein Data Bank (https://www.rcsb.org), UniProt Protein Database (https://www.uniprot.org/), National Center for Biotechnology Information Protein Database (https://www.ncbi.nlm.nih.gov/protein/), and Protein Data Bank (www.wwpdb.org), among others, MITF and TRP-1 were selected for docking simulations, which were conducted using Molecular Operating Environment software (https://www.chemcomp.com/en/Products.htm) and visualized using the PyMOL molecular visualization system (https://github.com/schrodinger/pymol-open-source).

### Molecular dynamics simulation and SPR analysis of the active components of RS-EVLP with MITF and TRP-1

To explore the interactions between the proteins MITF and TRP-1 and the ligands procyanidins and luteolin, molecular dynamics simulations of the protein-ligand complexes were performed using GROMACS 2020 (https://manual.gromacs.org/). Based on the AMBER99SB-ILDN force field, we constructed the protein topology and set up a cubic water box with a buffer distance of 1.0 nm (SPC216 water model). We then optimized the system energy using the steepest descent method after charge neutralization with Cl^-^/Na^+^ counterions. A 100-ps NVT (canonical ensemble, constant number of particles, volume, and temperature)/NPT (isothermal-isobaric ensemble, constant number of particles, pressure, and temperature) pre-equilibration (300 K, 1 bar) was then performed, followed by a production simulation for 30 ns (V-rescale temperature control, Parrinello-Rahman pressure control, and an integration step of 2 fs). Particle Mesh Ewald calculations were used to determine long-range electrostatic interactions (0.16-nm spacing), and the LINear Constraint Solver algorithm was used to calculate bond lengths. The trajectories were analyzed using VMD (Visual Molecular Dynamics) 1.9.3 (https://www.ks.uiuc.edu/Research/vmd/vmd-1.9.3/) and PyMOL 2.4.1 (https://pymol.org/), and the binding free energies were calculated using gmx_mmpbsa (https://valdes-tresanco-ms.github.io/gmx_MMPBSA/dev/). The SPR technique was used to study biomolecular interactions and analyze the affinity, kinetics, and other parameters of the binding of luteolin/procyanidin to MITF/TRP-1 by detecting changes in optical signals of biomolecules during binding and dissociation on the chip surface.

### Cell viability assay and internalized uptake of RS-EVLP

B16 cells (obtained from Shanghai Jisidenuo Biotechnology); RRID: (CVCL_F936); synonyms: (B16 sunline B78) in the logarithmic growth stage were inoculated into the wells of 96-well plates, which included blank controls (medium), negative controls (untreated cells), and the experimental group (RS-EVLP concentration gradient: 4.6 × 10^-4^ to 4.6 × 10^1^ µg/mL, “µg” refers to the protein content in the sample). Cells were cultured in Dulbecco’s modified Eagle’s medium [DMEM; (Thermo Fisher Scientific Inc, C11095500BT)] supplemented with 10% fetal bovine serum [FBS; (Zeta Life, Z7185FBS-500)] and 1% penicillin-streptomycin (Thermo Fisher Scientific Inc, 15140122) at 37 °C in a humidified atmosphere containing 5% CO_2_. After incubation for 24, 48, or 72 h, CCK-8 reagent (10 μL) was added to each well, and the plates were incubated for a further 2 h at 37 °C in the dark. The optical density (OD) at 450 nm was then detected using an enzyme marker^[39]^. Cell viability was calculated as (OD treated group - OD blank group)/(OD control group - OD blank group) × 100%.

DiI dye was mixed at a PBS:RS-EVLP:DiI ratio of 950:50:5 (v/v/v), protected from light, and incubated in a metal bath at 37 °C for 30 min. The mixture was then purified three times by ultracentrifugation (wX + ULTRA) at 100,000 × *g* for 70 min at 4 °C. B16 cells were inoculated into Corning CellBIND® dishes at a density of 5 × 10^4^ cells per dish. When cell fusion reached 50%, the cells were treated with 4.6 μg/mL DiI-labelled RS-EVLP for 2, 4, or 8 h. The nuclei and cytoskeleton were subsequently stained with fluorescein isothiocyanate and 4’,6-diamidino-2-phenylindole, respectively. Then, the cells were fixed with 4% paraformaldehyde for 15 min before quantitative analysis of the time-dependence of RS-EVLP, which was performed using three-channel imaging with a confocal microscope (Thunder DMi8; Leica Microsystems GmbH, Wetzlar, Germany)^[40,41]^.

### Measurement of relative melanin content and TYR activity

B16 melanoma cells (1 × 10^5^) were inoculated into the wells of 6-well plates containing Roswell Park Memorial Institute 1640 complete medium. After 24 h, the culture medium was replaced with basic complete DMEM and the cells were incubated with RS-EVLP, along with 200 ng/mL of α-MSH (MedChemExpress, Monmouth Junction, NJ, USA) and 1,500 nM/mL of arbutin (Shanghai Macklin Biochemical Technology Co., Ltd., Shanghai, China), at protein concentrations of 4.6 and 23 μg/mL, respectively. After 48 h, the cells were washed twice with PBS, examined under a microscope for melanin deposition phenotypes, and the solution was discarded. Then, 1 M NaOH (800 μL) was added to each well, and the solution was lysed at 80 °C for 1 h.

The lysates were transferred to the wells of a 96-well plate, and the relative melanin content was determined by measuring the absorbance at 405 nm using an enzyme marker^[42]^. Equal numbers of treated cells were lysed with 1 mL of 1% Triton X-100, subjected to a freeze-thaw cycle at -80 °C (30 min → 10 min at room temperature), and then centrifuged at 12,000 × *g* for 15 min. The resulting supernatant was incubated with 2 mg/mL of L-3,4-dihydroxyphenylalanine (L-DOPA) substrate at 37 °C for 1 h. Afterward, the absorbance was measured at 475 nm to detect TYR activity.

### Reverse transcription quantitative polymerase chain reaction (RT-qPCR)

Total RNA was extracted from B16 cells that had been treated for 48 h using TRIzol Reagent (Ambion®; Thermo Fisher Scientific) and reverse-transcribed into complementary DNA using 5× PrimeScript RT Master Mix (TaKaRa Bio, Inc., Shiga, Japan). The RT-qPCR assays were performed using 2× UltraSYBR® Green Mix (Beijing Kangwei Century Biotechnology Co., Ltd., Beijing, China) on a real-time fluorescence quantitative PCR analyzer (Thermo Fisher Scientific). β-actin was used as the internal reference gene. The transcriptional regulatory effects of MITF, TYR, and TRP-1/TRP-2 were quantitatively analyzed in control cells and groups treated with α-MSH (200 ng/mL), arbutin (1,500 nM/mL), and RS-EVLP (4.6/23 μg/mL)^[43]^ using the 2^-ΔΔCt^ method with the primer sequences listed in Table 1.

**Table 1:** Primer sequences used for RT-qPCR

**Gene**	**Primer**	**Sequence**
*m-MITF*	Forward	CAAATGGCAAATACGTTACCCG
	Reverse	CTCCCTTTTTATGTTGGGAAGGT
*m-TYR*	Forward	CTCTGGGCTTAGCAGTAGGC
	Reverse	GCAAGCTGTGGTAGTCGTCT
*m-TRP-1*	Forward	CCCCTAGCCTATATCTCCCTTTT
	Reverse	TACCATCGTGGGGATAATGGC
*m-TRP-2*	Forward	TTCTGCTGGGTTGTCTGGG
	Reverse	CACAGATGTTGGTTGCCTCG
*β-actin*	Forward	GGCTGTATTCCCCTCCATCG
	Reverse	CCAGTTGGTAACAATGCCATGT

RT-qPCR: Reverse transcription quantitative polymerase chain reaction.

### Enzyme-linked immunosorbent assay (ELISA)

Drug-treated adherent cells were collected and washed with pre-cooled PBS before digestion with 0.25% trypsin containing ethylenediaminetetraacetic acid. The cell precipitate was collected by centrifugation. The freeze-thaw lysis method was then employed: the cell precipitates (1 × 10^6^ cells) were resuspended in 150 μL of PBS, centrifuged at 3,000 rpm for 5 min at 4 °C, and underwent three freeze-thaw cycles (-20 °C/30 min → 37 °C/10 min). The resulting supernatant was then extracted by centrifugation at 15,000 × *g* for 10 min at 4 °C. Key regulatory proteins of melanin synthesis were detected using a mouse MITF/TYR/TRP-1/TRP-2 ELISA kit (Beijing Dongge Boye Biotechnology, DG96647Q/DG96642Q/DG30784M/DG96648Q), and protein concentrations were calculated based on standard curves.

### Zebrafish *in vivo* studies

Experiments were performed using wild-type AB strain zebrafish embryos (Hangzhou Hunter Biotechnology Co., Ltd., Beijing, China). Embryos at 24 h post-fertilization (hpf) were inoculated into the wells of 6-well plates at a density of 15 embryos per well. The embryos were then grouped and treated as follows: a blank control group (standard zebrafish culture); a positive control group (1 mg/mL arbutin); and an experimental group (RS-EVLP gradient at concentrations of 0.046/0.23/0.46 mg/mL). The embryos in each group were exposed to the treatment for 24, 48, or 72 h at a constant temperature of 28.5 °C under a 14/10-h light/dark cycle. The culture medium containing the corresponding concentration of the treatment reagents was replaced every 24 h. Each experiment was performed with *n* = 15 zebrafish larvae per group. All animal experiments were approved by the Animal Ethics Committee of Ruiye Model Animal (Guangzhou) Biotechnology Co., Ltd. (ethical approval no. RYEth-20250627752).

### Photography of zebrafish body surface pigmentation

MS-222 anesthetic (Hangzhou Jiuyan Biotechnology Co., Ltd., Hangzhou City, China) was diluted 100-fold in 6% methylcellulose (Shanghai Yuanye Bio-Technology Co., Ltd., Shanghai, China) and used as the fixation matrix after vortexing. Zebrafish larvae at 24/48/72 hpf were treated, placed on concave slides pre-set with fixative, and imaged *in vivo* using an MZ62 somatic microscope (10× objective). The grey scale values of the pigmented region of the zebrafish heads were analyzed quantitatively using ImageJ software (https://imagej.net/ij/), and the survival rate of juvenile zebrafish in each group was calculated^[44]^.

### Zebrafish melanin assay

Groups of zebrafish embryos at 24 hpf were exposed to basal culture medium, 1 mg/mL of arbutin, and gradient concentrations of RS-EVLP (0.046, 0.23, and 0.46 mg/mL) for 72 h. The embryos were then quickly frozen and ground in liquid nitrogen. The tissue homogenate was then centrifuged at 10,000 × *g* for 15 min at 4 °C, and the precipitate was lysed by adding 1 M NaOH containing 10% dimethyl sulfoxide (500 μL per sample) at 80 °C for 1 h. The lysate was transferred to the wells of 96-well plates, and the OD at 405 nm was detected using an enzyme marker. The relative melanin content of each group (%) was calculated as the OD value of that group/OD value of the negative control group × 100%^[45]^.

#### Skin transdermal absorption assay

The transdermal properties of the RS-EVLP were evaluated using the DiI fluorescent labeling method. RS-EVLP were incubated with DiI dye at an optimized ratio (PBS:RS-EVLP:DiI = 950:50:9) in the presence of light at 37 °C for 30 min. The labeled complexes were obtained by three rounds of purification by ultracentrifugation at 100,000 × *g* for 70 min at 4 °C. The DiI-RS-EVLP complexes were loaded onto a mask cloth and applied to the back skin of anesthetized nude mice. Skin samples were obtained at 15, 30, and 60 min, respectively, fixed with 4% paraformaldehyde, embedded in paraffin wax, and cut into 5 µm-thick sections. The temporal osmotic gradient from the epidermis to the dermis was analyzed by quantifying the exposure time using a confocal fluorescence microscope.

#### Clinical assessment: phase I trial of RS-EVLP to improve skin pigmentation

This randomized, self-controlled clinical trial was approved by the Ethics Committee of the Third Affiliated Hospital of Guangzhou University of Chinese Medicine (approval No. PJ-XS-20230919-002) and was conducted in accordance with the principles of Good Clinical Practice and relevant regulatory requirements. Written informed consent was obtained from all the participants. This clinical part is an exploratory cosmetic trial. In total, 10 healthy adult volunteers (age ≥ 18 years) who met strict inclusion/exclusion criteria were screened to evaluate the whitening efficacy of a 5% formulation of RS-EVLP prepared in purified water, glycerol, and nitrone.

Inclusion criteria: (1) Presence of melasma, freckles, post-inflammatory hyperpigmentation (e.g., acne-induced), seborrheic keratosis, or other types of hyperpigmentation; (2) Age between 18 and 70 years; (3) No use of other topical whitening agents or laser therapy within 1 month prior to treatment; (4) Ability to understand and willingness to participate in this clinical study, with good compliance.

Exclusion criteria: (1) Women who are pregnant or breastfeeding; (2) Presence of infectious or contagious rash at the treatment site; (3) Patients with sensitive skin who are prone to allergies following topical application; (4) Patients who have received or may be exposed to intense sunlight exposure within one month after treatment; (5) Individuals with photosensitive skin diseases or those who have recently taken photosensitizing drugs such as oral retinoids.

Experimental protocol: A split-design approach was employed, in which test preparation was applied to the dorsal aspect of one hand, the carrier control was applied to the dorsal aspect of the opposite hand, and hydroquinone cream (0.1 g) was applied to the wrist as a positive control. The preparations were applied twice daily (morning and evening) at fixed intervals, followed by a 15-min exposure time. Then, the area was rinsed. The participants were instructed to avoid using other cosmetic products during the 3-week study period.

Assessment method: L^*^ values (luminance, with values between 0 and 100, where 0 indicates black and 100 indicates white) were measured using a dermatoscope to quantitatively assess skin brightness before the test and at 1, 2, and 3 weeks afterward^[46]^.

#### Statistical analysis

All experiments were performed with at least three independent biological replicates, unless otherwise specified. Biological replicates are defined as independent samples or repeated experiments, and their sample sizes (*n* values) are indicated in the respective figure legends. Technical replicates for all assays (including cell-based, zebrafish, and clinical studies) were performed with three repeated measurements per biological sample (*n* = 3 for technical replicates), and the average values were used for statistical analysis. Results are expressed as mean ± standard deviation (SD) or mean ± standard error of the mean (SEM), as indicated in the figure legends. Statistical comparisons between groups were performed using one-way analysis of variance (ANOVA) followed by Tukey’s post-hoc test or Dunnett’s multiple comparisons test, or repeated-measures two-way ANOVA followed by Bonferroni’s post-hoc test, as appropriate. A *P*-value < 0.05 was considered statistically significant. All statistical analyses were performed using GraphPad Prism.

## RESULTS

### Extraction and characterization of RS-EVLP

RS-EVLPs were isolated from rose petals by ultracentrifugation [Figure 1A] and observed by transmission electron microscopy, which revealed a double-layered, elliptical structure of various sizes [Figure 1B]. Protein analysis revealed a molecular weight of 25-35 kDa, while nanoparticle tracking analysis showed a particle size distribution of 70-100 nm. Membrane integrity was confirmed by treatment with Triton X-100, with an increased detergent concentration indicating a higher degree of vesicle cleavage and purity of up to 83% [Figure 1C-E]. Metabolomics analysis revealed that the RS-EVLP contained high proportions of lipids and lipid-like molecules (38%), followed by organic acids (14%) and phenylpropanoids (9%). These compounds are involved in plant defense and signaling. Other components included phenylpropanoids and polyketides (9%), which have antioxidant, anti-inflammatory, antibacterial, and antiviral properties. Further analysis of lipid subclasses revealed 20% fatty acids, 15% glycerophospholipids, and 13% isoprenoid lipids and flavonoids (with antioxidant properties) [Figure 1F and G]. Notably, RS-EVLP contains six bioactive compounds abundant in roses: quercetin, luteolin, apigenin, kaempferol, ellagic acid, and procyanidin B3 [Figure 1H and I].

**Figure 1:**
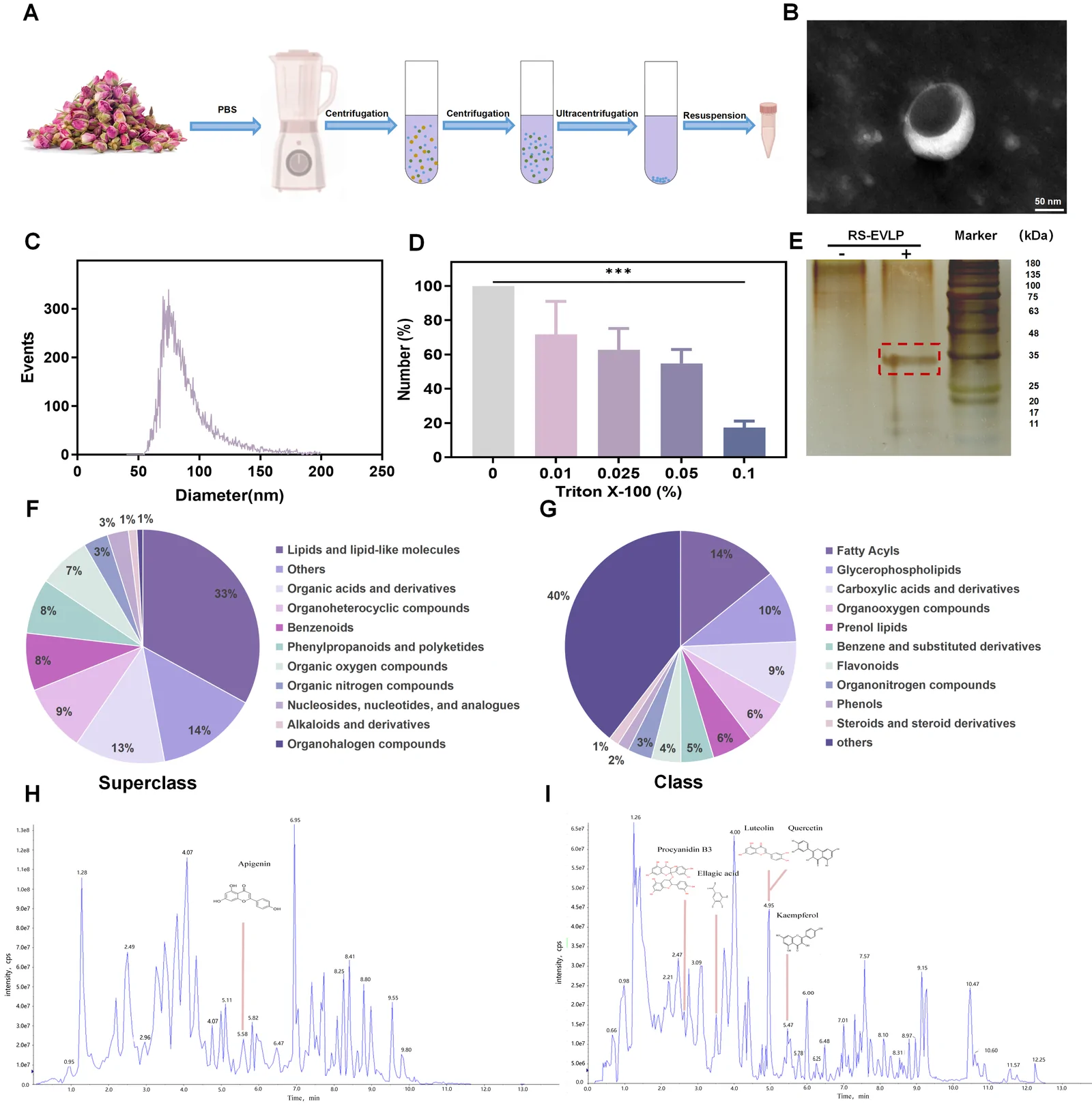
Extraction and identification of RS-EVLP. (A) Extraction of RS-EVLP from roses by ultrafast differential centrifugation; (B) TEM to observe the RS-EVLP morphology and size, with a 50-nm scale bar; (C) Nano-flow cytometry to detect the RS-EVLP particle size distribution; (D) Triton X-100 to detect the lipid membrane structure of the RS-EVLP; (E) Protein electrophoresis to identify the RS-EVLP proteins; (F and G) RS-EVLP metabolite species; (H and I) Mass spectrometry to analyze the positive and negative ion flow patterns of the RS-EVLP. Data are presented as mean ± SEM. Statistical significance was determined by one-way ANOVA followed by Tukey’s post-hoc test: ^***^P < 0.001. ANOVA: Analysis of variance; RS-EVLP: rose-derived extracellular vesicle-like particles; SEM: standard error of the mean; TEM: transmission electron microscopy.

### Association of RS-EVLP metabolites with pigmentation disease-related component targets

The results show that the 47 active RS-EVLP compounds are linked to hyperpigmentation, with a subset of 159 gene targets. The quantitative relationships are presented as a Venn diagram [Figure 2A and B]. Additionally, the interactions among the active compounds of the RS-EVLP and the hyperpigmentation-related intersecting targets were demonstrated with a network pharmacology map and a protein-protein interaction network [Figure 2C and D]. The active compounds of the RS-EVLP are linked to the melanogenesis-related biological pathway [Figure 2E]. GO analysis revealed various molecular functions associated with RS-EVLP, including protein kinase activity, growth factor receptor binding, and oxidoreductase activity [Figure 2F].

**Figure 2:**
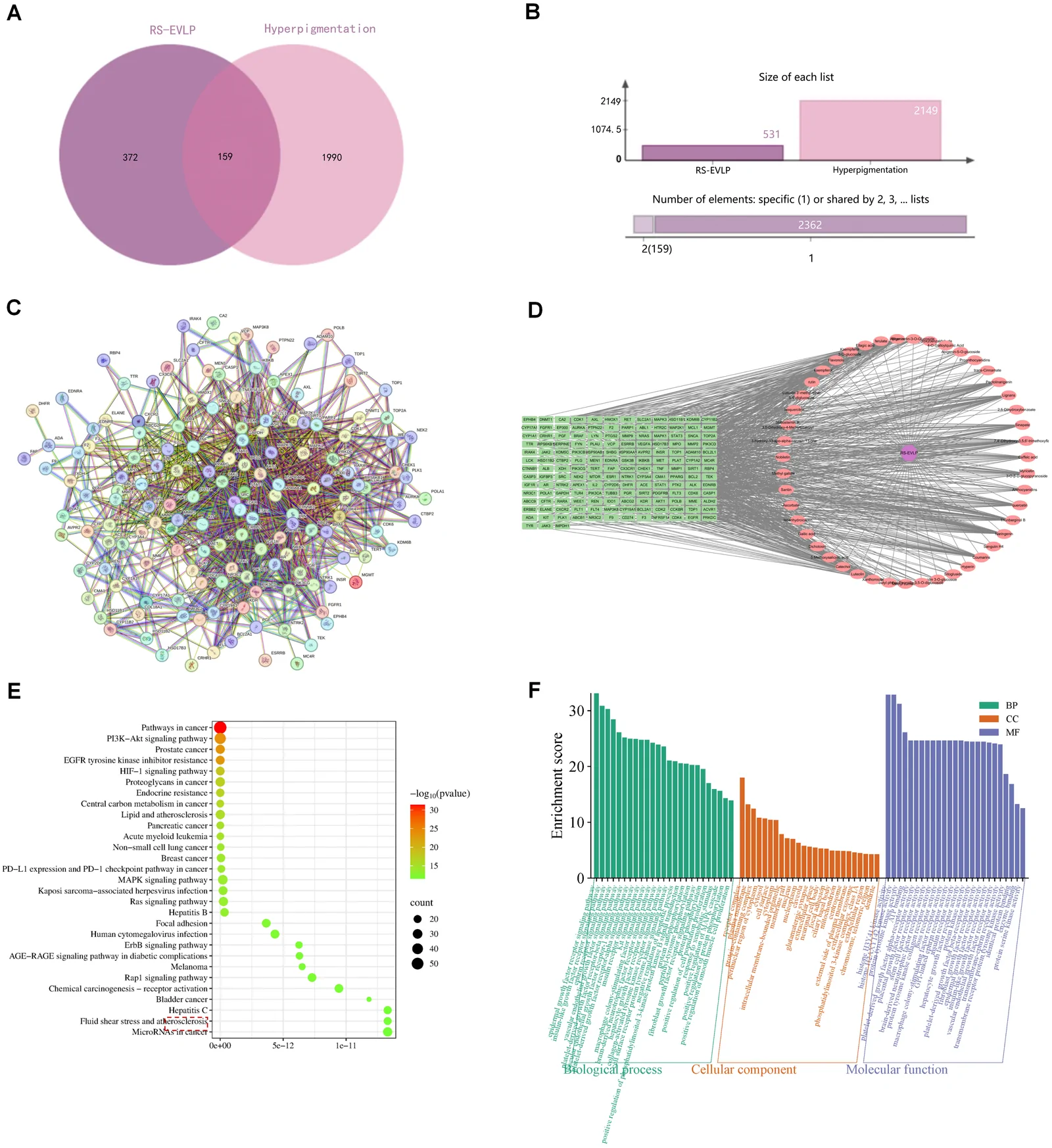
Network pharmacological mechanism study of RS-EVLP for hyperpigmentation. (A and B) Venn diagram of active ingredient targets and genes involved in hyperpigmentation, as well as intersections; (C) Protein-protein interaction network of core targets; (D) Network pharmacology map showing the intersection of RS-EVLP ingredient targets and pigmentation disease targets; (E) KEGG pathway enrichment analysis demonstrating the key signaling pathways; (F) GO function enrichment analysis. GO: Gene Ontology; KEGG: Kyoto Encyclopedia of Genes and Genomes; RS-EVLP: rose-derived extracellular vesicle-like particles.

### Molecular docking results of RS-EVLP key compounds

The results showed that quercetin, luteolin, apigenin, kaempferol, ellagic acid, and procyanidins could all bind to the allosteric sites of MITF and TRP-1 proteins, with predicted binding energies of -6.22, -5.06, -5.08, -4.73, -4.98, and -4.90 for MITF, and -5.85, -5.84, -5.60, -5.58, -5.69, and -7.47 for TRP-1, respectively. This indicates that the luteolin and procyanidins in RS-EVLP can form strong binding affinities with the allosteric sites of the MITF and TRP-1 proteins [Figure 3A-D and Supplementary Figure 1A-K]. It is hypothesized that these active RS-EVLP ingredients can inhibit melanogenesis by binding to the amino acid residues of the MITF and TRP-1 proteins.

**Figure 3:**
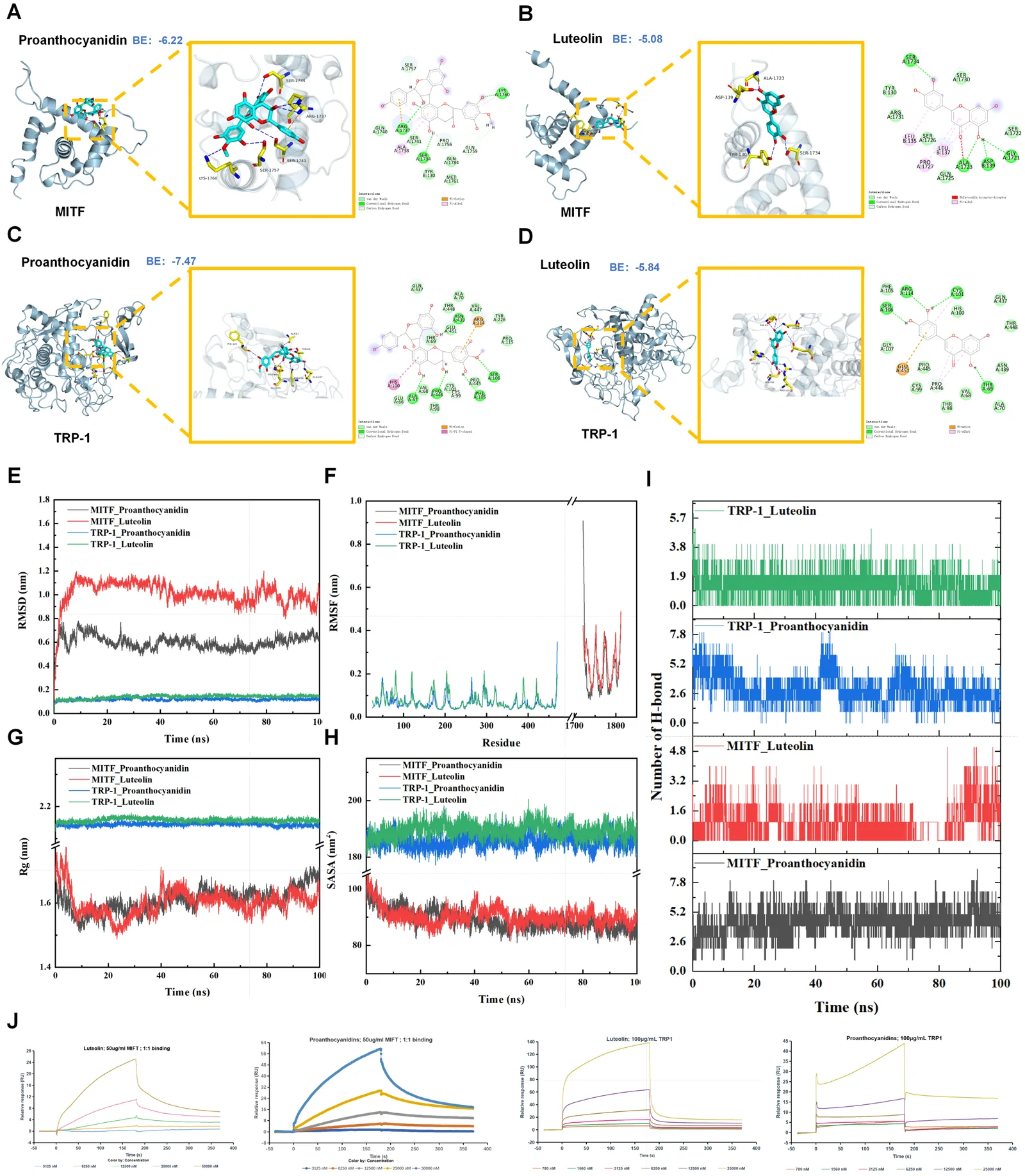
The interaction mechanism of the key active components of RS-EVLP and target proteins, including molecular docking, molecular dynamics simulation, and SPR validation. (A-D) Molecular docking patterns of procyanidins and luteolin with the key binding sites of MITF and TRP-1 proteins, respectively; (E) RMSD of proteins during simulation of protein-ligand complexes over time; (F) RMSF of the protein-ligand complex simulation process over time; (G) RA curve of proteins during protein-ligand complex simulation *vs*. time; (H) SASA curve of proteins during protein-ligand complex simulation *vs*. time; (I) Number of hydrogen bonds curve during protein-ligand complex simulation *vs*. time; (J) SPR binding kinetics curves of procyanidins, luteolin, and MITF and TRP-1 proteins. RA: Radius of gyration; RMSD: root mean square deviation; RMSF: root mean square fluctuation; RS-EVLP: rose-derived extracellular vesicle-like particles; SASA: solvent-accessible surface area; SPR: surface plasmon resonance; MITF: microphthalmia-associated transcription factor; TRP-1: tyrosinase-related protein 1.

### Results of molecular dynamics simulation and SPR analysis

The network pharmacological analysis (Section “Molecular docking results of RS-EVLP key compounds”) and molecular docking results revealed an association of RS-EVLP with the melanogenic pathway. Two sets of complexes with stronger binding energy (procyanidins/luteolin-MITF/TRP-1) were screened for validation. We found that procyanidins bind to MITF and TRP-1 proteins more stably, as shown by lower RMSD values, higher structural tightness (lower “solvent accessible surface area” and “radius of gyration” values), fewer flexibility changes (lower “root mean square fluctuation” values), and more hydrogen bonds [Figure 3E-H]. Binding free energy analysis showed that procyanidins had lower binding energies (mainly dependent on van der Waals forces), whereas luteolin was weakly bound [Figure 3I]. Procyanidins were also expressed at high abundance in RS-EVLP, suggesting that they influence melanogenesis by stabilizing the protein structure and providing theoretical support for the potential application of RS-EVLP. SPR analysis showed that both procyanidins and luteolin specifically bound to MITF and that the binding process was consistent with the 1:1 model. These results provide a quantitative basis to investigate the roles of procyanidins and luteolin in biological processes, such as melanogenesis [Figure 3J].

### Uptake and absorption of RS-EVLP by B16 cells

To assess the interaction between RS-EVLP and target cells, we first confirmed that RS-EVLP did not exhibit an antiproliferative effect on B16 cells at concentrations below 46 µg/mL. Two non-cytotoxic concentrations were therefore selected for uptake studies: 4.6 and 23 µg/mL. Fluorescein-labeled RS-EVLP exhibited time- and dose-dependent cellular uptake in B16 cells. Flow cytometry showed maximum cellular uptake (94.76% positive cells) at 23 µg/mL after 8 h. Confocal microscopy further confirmed that the RS-EVLPs were efficiently taken up into the cytoplasm of B16 cells, with fluorescence intensity increasing proportionally with incubation time [Figure 4A-C].

**Figure 4:**
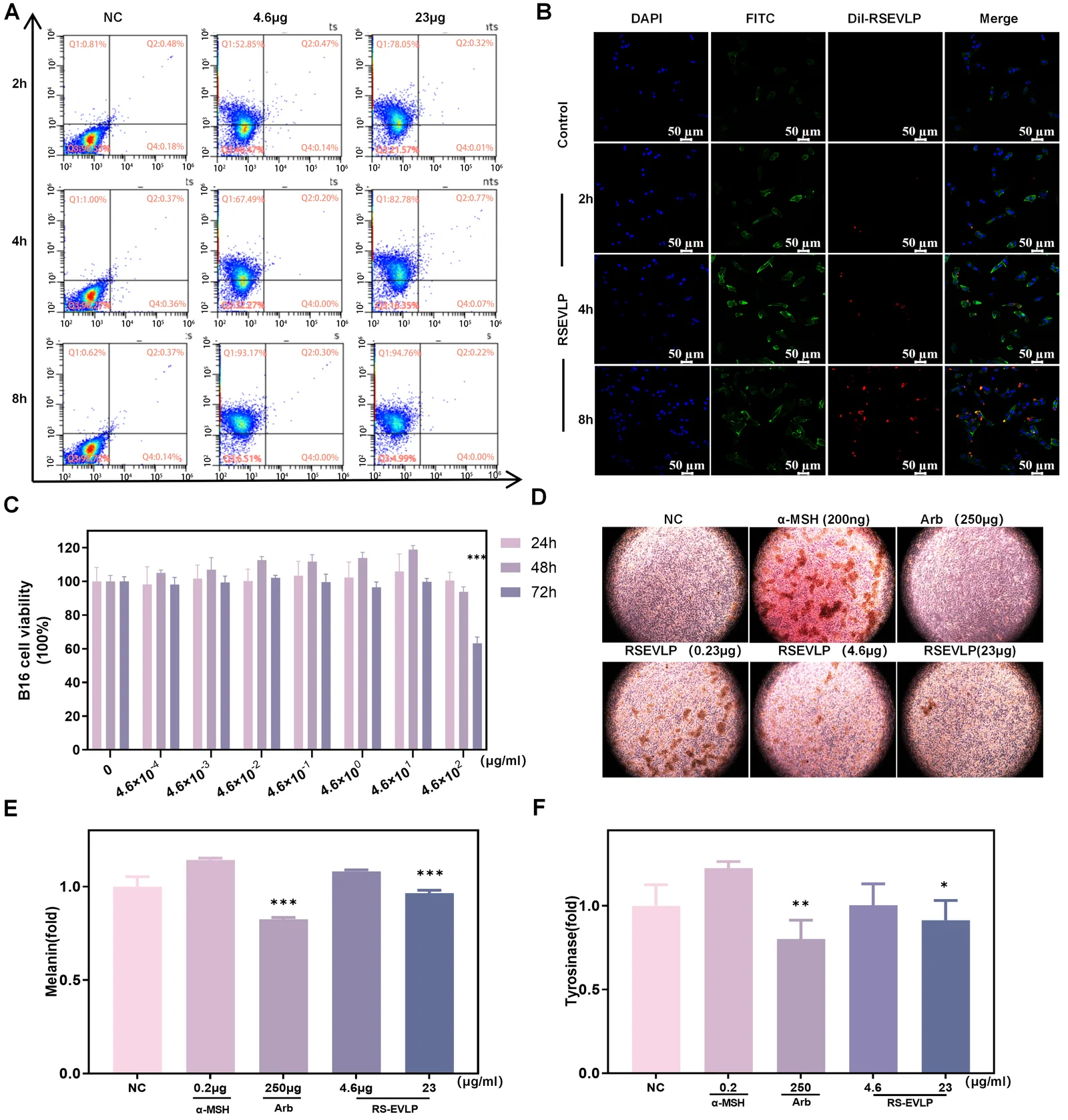
Evaluation of the uptake, cell viability effect, and anti-pigmentation efficacy of RS-EVLP in B16 mouse melanoma cells. (A) Quantitative flow cytometry analysis of B16 cell uptake after treatment with different RS-EVLP concentrations (4.6 and 23 µg/mL) for different times (2, 4, and 8 h); (B) Fluorescence microscopy images (scale bar: 50 µm) of B16 cell uptake after treatment with different RS-EVLP concentrations (4.6 and 23 µg/mL) of RS-EVLP for different times (2, 4, and 8 h); (C) The effect of different RS-EVLP concentrations on B16 cell viability; (D) Morphological microscopic images of melanogenesis in B16 cells stimulated by α-MSH, the positive control Arb, and RS-EVLP treatments at different concentrations (0.23, 4.6, and 23 µg/mL); (E and F) Quantitative analysis of (E) melanin content and (F) tyrosinase activity in B16 cells under the same treatment conditions. Data are shown as mean ± SEM from three independent biological replicates (*n* = 3). Statistical significance was determined by one-way ANOVA followed by Tukey’s multiple comparisons test (*vs*. control group): ^*^P < 0.05; ^**^P < 0.01; ^***^P < 0.001. ANOVA: Analysis of variance; Arb: arbutin; α-MSH: α-melanocyte-stimulating hormone; DAPI: 4’,6-diamidino-2-phenylindole; FITC: fluorescein isothiocyanate; NC: negative control; RS-EVLP: rose-derived extracellular vesicle-like particles; SEM: standard error of the mean.

### RS-EVLP inhibited α-MSH-induced melanogenesis and TYR activity in B16 cells

B16 cells were stimulated with 200 ng/mL of α-MSH and then treated with various concentrations of RS-EVLP (0.23, 4.6, 10, 20, and 23 µg/mL) or arbutin (250 µg/mL; positive control). After 48 h, microscopic analysis revealed that α-MSH had successfully induced melanin synthesis, whereas RS-EVLP showed an inhibitory effect at 4.6 and 23 µg/mL, as well as arbutin (positive control) at 250 µg/mL. After 48 h, microscopic analysis showed that α-MSH had successfully induced melanin synthesis, whereas both arbutin and RS-EVLP (4.6 and 23 µg/mL) significantly reduced melanin synthesis as compared to the cell group treated with α-MSH only [Figure 4D]. The NaOH assay showed that the inhibitory effect of the RS-EVLP on melanogenesis was concentration-dependent, with the highest concentration (23 µg/mL) having the most significant inhibitory effect (16% reduction in melanin content; Figure 4E). Furthermore, the RS-EVLP significantly inhibited TYR activity, as demonstrated by the L-DOPA oxidase assay (18% and 26% reductions in the 4.6 and 23 µg/mL groups, respectively; Figure 4F). This is consistent with the inhibitory effect on melanogenesis, suggesting that the RS-EVLP may affect the melanin synthesis pathway by modulating TYR activity.

### RS-EVLP regulated the expression of genes and proteins related to melanogenesis

In order to elucidate the molecular mechanism employed by the RS-EVLP to regulate melanogenesis, B16 cells induced by α-MSH were treated with 23 µg/mL of RS-EVLP for 48 h. Total RNA was then extracted with TRIzol reagent [Figure 5A and B]. Melanin synthesis is closely linked to MITF, the ultimate target of multiple signaling pathways, and TYR, a key enzyme in melanogenesis. Melanin synthesis mainly involves three enzymes in the TYR gene family: TYR, TRP-1, and TRP-2. We therefore examined the mRNA expression of MITF, the core regulator of melanin synthesis, and related downstream effector molecules (TYR, TRP-1, and TRP-2). The experimental results showed that treatment with RS-EVLP at 23 µg/mL significantly suppressed the expression of these genes compared with the α-MSH-induced control group [Figure 5C-F]. Further examination of protein levels with an ELISA revealed that RS-EVLP reduced expression of MITF, TYR, TRP-1, and TRP-2 in a concentration-dependent manner. In the 4.6 µg/mL treatment group, the protein levels of MITF, TYR, TRP-1, and TRP-2 were reduced by 30%, 18%, 25%, and 29%, respectively, while in the 23 µg/mL treatment group, the reduction was even more significant at 41%, 40%, 44%, and 47%, respectively [Figure 5G-J]. These findings suggest a potential mechanism by which RS-EVLP regulates melanogenesis.

**Figure 5:**
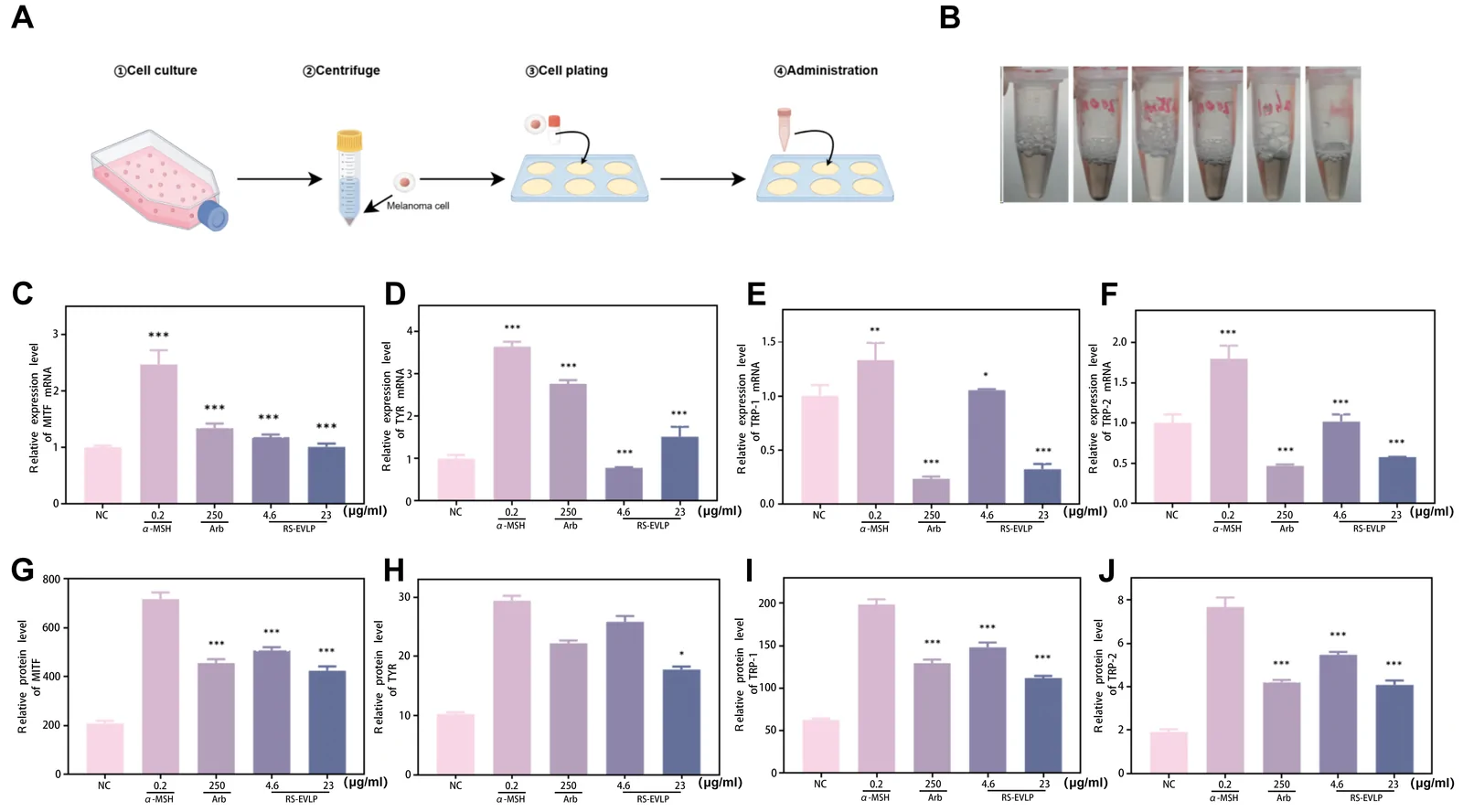
Regulatory effects of RS-EVLP on the expression of key melanogenesis pathway genes and proteins in B16 cells. (A) Cells were cultured and treated with compounds (α-MSH, arbutin, or RS-EVLP at 4.6 and 23 µg/mL); (B) Representative photographs of cell pellets harvested from each treatment group; (C-F) RT-qPCR analysis of the effect of different concentrations of RS-EVLP on the expression of coregulated genes (4.6 and 23 µg/mL) of RS-EVLP-treated B16 cells for 48 h; (G-J) ELISA analysis of the regulatory effects of RS-EVLP on key proteins [(G) MITF; (H) TYR; (I) TRP-1; and (J) TRP-2]. Data are expressed as the mean ± SEM from three independent biological replicates (*n* = 3). Statistical significance was determined by one-way ANOVA followed by Tukey’s multiple comparisons test (*vs*. control group): ^*^P < 0.05; ^**^P < 0.01; ^***^P < 0.001. ANOVA: Analysis of variance; α-MSH: α-melanocyte-stimulating hormone; ELISA: enzyme-linked immunosorbent assay; MITF: microphthalmia-associated transcription factor; RS-EVLP: rose-derived extracellular vesicle-like particles; RT-qPCR: reverse transcription quantitative polymerase chain reaction; SEM: standard error of the mean; TRP-1: tyrosinase-related protein 1; TRP-2: tyrosinase-related protein 2; TYR: tyrosinase.

### The anti-melanogenic effect of RS-EVLP on zebrafish embryos

This study evaluated the effect of RS-EVLP on melanogenesis *in vivo* using a wild AB zebrafish embryo model. Different concentrations of RS-EVLP (0.046, 0.23, and 0.46 mg/mL) and a positive control (arbutin at 1 mg) were administered to observe changes to body pigmentation at 24, 48, and 72 h. *In vivo* microscopic observation showed that RS-EVLP attenuated body surface pigmentation in a concentration-dependent manner [Figure 6A]. Subsequently, zebrafish larvae at 72 h post-treatment were subjected to tissue homogenization, and the relative melanin content was determined using the NaOH method. ImageJ quantitative analysis confirmed that the melanin content in the head was significantly lower in each RS-EVLP-treated group than the NC group [Figure 6B and C]. Notably, none of the tested concentrations exhibited embryotoxicity. The NaOH assay further showed that arbutin reduced melanin content by 15.1%, whereas RS-EVLP at 0.046, 0.23, and 0.46 mg/mL resulted in reductions of 7.0%, 6.0%, and 10.7%, respectively, which were consistent with the phenotypic observations [Figure 6D-F].

**Figure 6:**
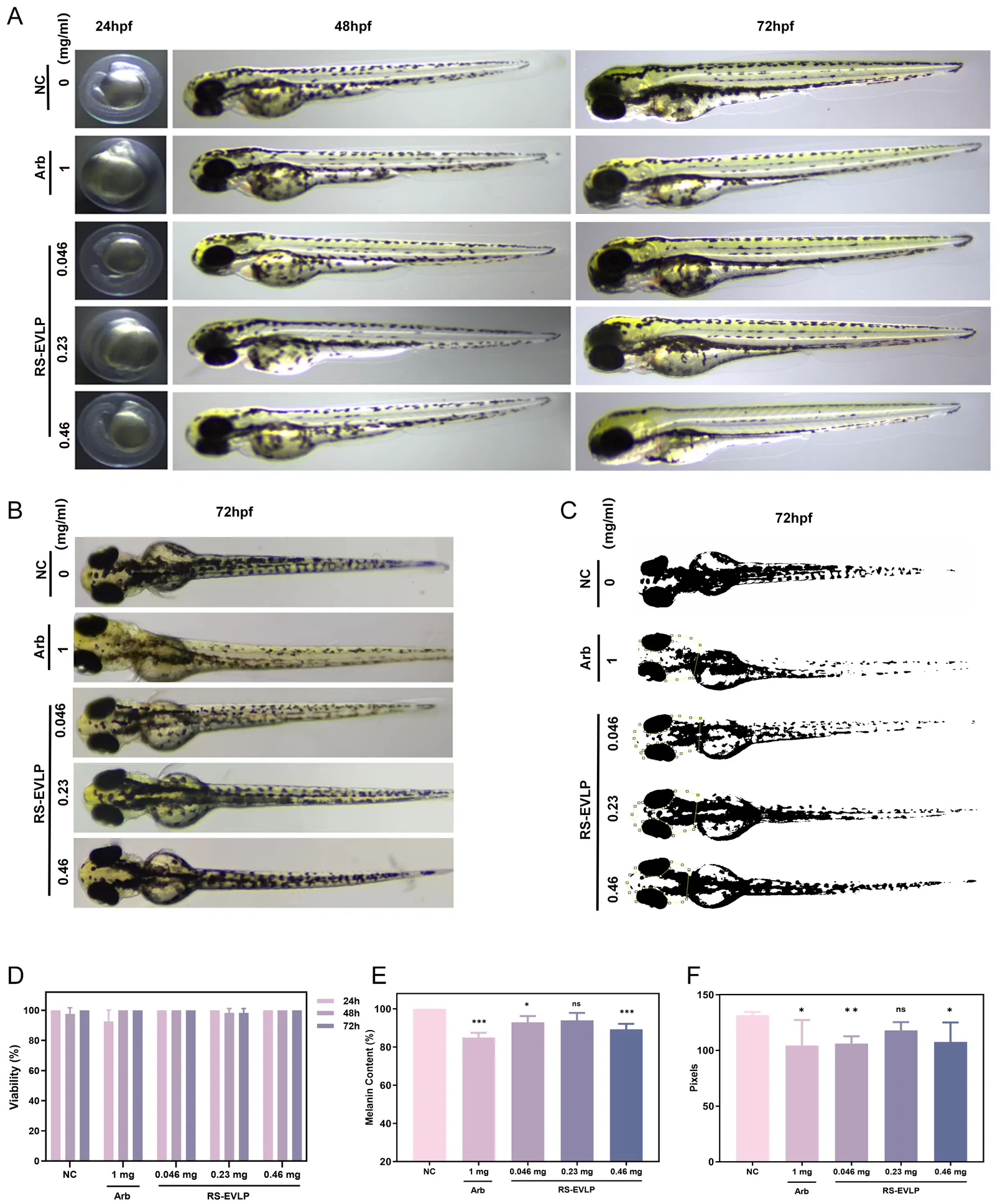
Antipigmentation Effect and Safety Evaluation of RS-EVLP in a Zebrafish Model. (A and B) Stereo microscope images of 24-hpf embryos treated with zebrafish culture medium (control group), arbutin (positive control group), and different concentrations of RS-EVLP (0.046, 0.23, and 0.46 mg/mL) of RS-EVLP for 24, 48, and 72 h; (C) ImageJ software analysis of zebrafish head pigmentation values (*n* = 6 biological replicates per group); (D) Zebrafish survival rates at 24, 48, and 72 h after RS-EVLP administration (*n* = 15 biological replicates per group); (E) Melanin content in zebrafish lysates after 72 h of treatment with different test substances, measured by OD405 (*n* = 15 biological replicates per group); (F) Quantitative analysis of melanin pigmentation intensity in the zebrafish head based on ImageJ software (*n* = 6 biological replicates per group). Data are shown as mean ± SEM from the indicated number of biological replicates, with each biological sample measured in triplicate (technical replicates). Statistical significance was determined by one-way ANOVA followed by Dunnett’s multiple comparisons test (*vs*. control group): ^*^P < 0.05; ^**^P < 0.01; ^***^P < 0.001. ANOVA: Analysis of variance; hpf: hours post-fertilization; OD: optical density; RS-EVLP: rose-derived extracellular vesicle-like particles; SEM: standard error of the mean.

### Skin transdermal absorption assay

Transdermal analysis was conducted to assess the penetration density of the DiI-RS-EVLP group compared to the DiI group over 15 min to 1 h. Permeation density was measured by integrating the fluorescence intensity, which reflects the distribution of DiI-labelled particles in the skin tissue of nude mice. DiI-labelled RS-EVLP showed time-dependent enhancement of skin permeation from 15 min to 1 h. Quantitative fluorescence analysis showed that the penetration density was higher in the RS-EVLP-treated group than in the DiI-only control group, with a progressive increase over time. This may be attributed to the phospholipid bilayer structure of the RS-EVLP, which promotes diffusion and penetration into the skin. The results showed that RS-EVLP exhibited strong penetration ability in skin tissue, entering the stratum corneum and gradually penetrating into the basal layer over time [Figure 7A-C].

**Figure 7:**
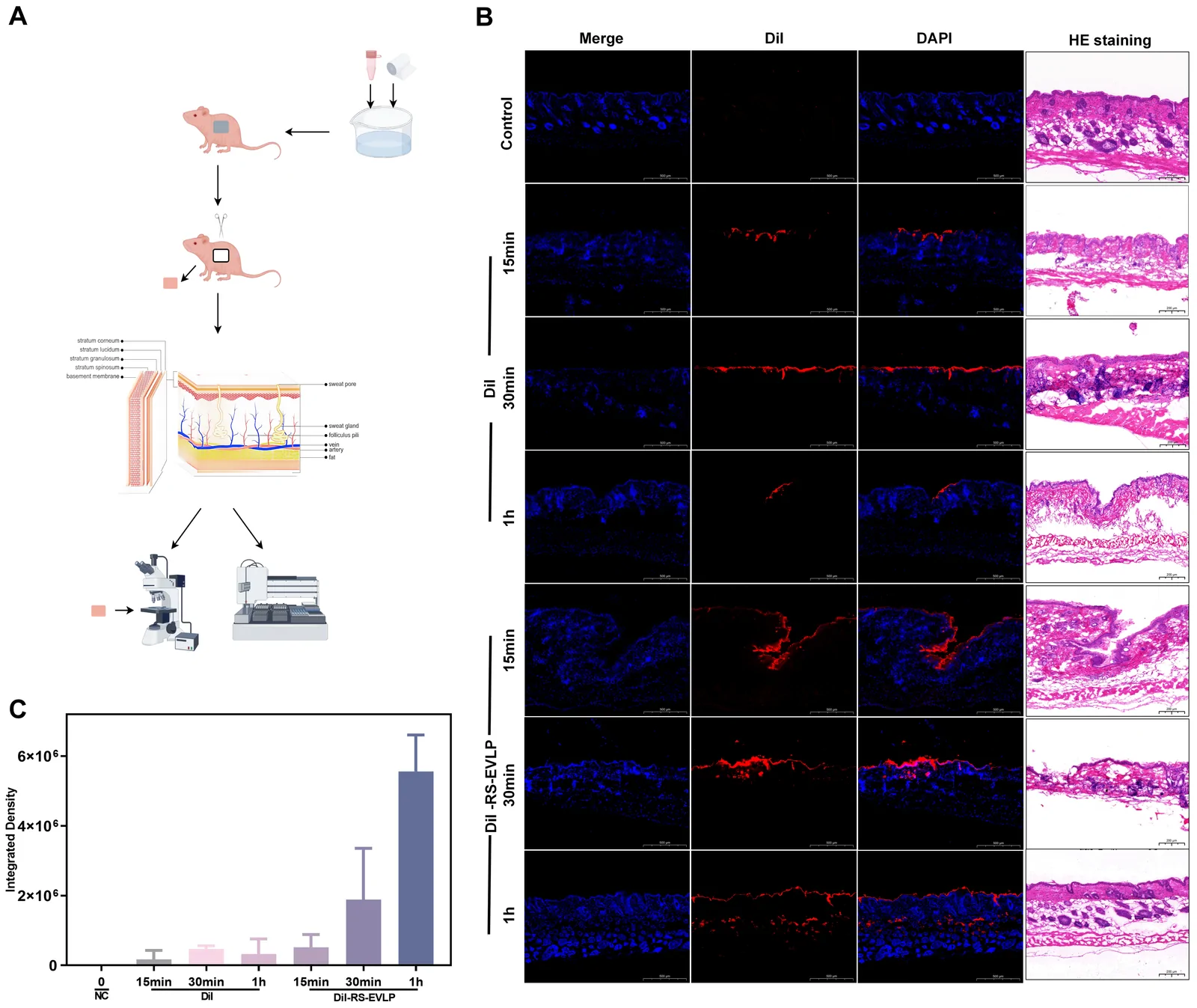
Evaluation of the transdermal permeation properties of the RS-EVLP complex in the epidermis of nude mice. (A) Transdermal experimental design flowchart; (B) Fluorescence micrographs showing the depth of stratum corneum penetration in the normal control group and DiI dye-treated groups (15, 30, and 60 min), as well as the DiI-labeled RS-EVLP complex-treated groups (15, 30, and 60 min); (C) Permeation density trends at different time points. Data are shown as mean ± SEM from *n* biological replicates (as indicated in each panel), with each biological sample measured in triplicate (technical replicates, *n* = 3). Note: The focus was on observing the efficacy of the RS-EVLP carrier itself. So, statistical analysis was not performed, as this experiment focused on qualitative observations of penetration-depth trends. Trend-change graphs of the penetration density of the treatment groups at different time points. DAPI: 4’,6-diamidino-2-phenylindole; DiI: 1,1’-dioctadecyl-3,3,3’,3’-tetramethylindocarbocyanine perchlorate; RS-EVLP: rose-derived extracellular vesicle-like particles; SEM: standard error of the mean.

### Clinical efficacy observations

Clinical evaluation showed that the 5% RS-EVLP preparation improved skin pigmentation. The L^*^ value of the RS-EVLP group increased steadily from day 8 (ΔL^*^ = 1.27, *P* = 0.0004) to day 21 (ΔL^*^ = 2.15, *P* < 0.0001), demonstrating a significantly greater whitening effect than hydroquinone cream (day 21, ΔL^*^ = 1.29, *P* = 0.0060). Notably, the RS-EVLP group exhibited a cumulative effect over time, with the difference in L^*^ values increasing from 1.27 to 2.15. This suggests that pigmentation regulation is achieved through multiple mechanisms, and that the product may be suitable for long-term management of pigmentation issues. Furthermore, the difference in L^*^ values between the treated and control surfaces of the RS-EVLP group widened over time (0.27 on day 8 to 2.15 on day 21) [Figure 8A and B], suggesting that a cumulative effect suitable for long-term pigmentation management, potentially circumventing the risk of pigment rebound associated with conventional chemical whiteners. Safety analysis showed no serious adverse reactions in any of the subjects, confirming that the RS-EVLP were well tolerated.

**Figure 8:**
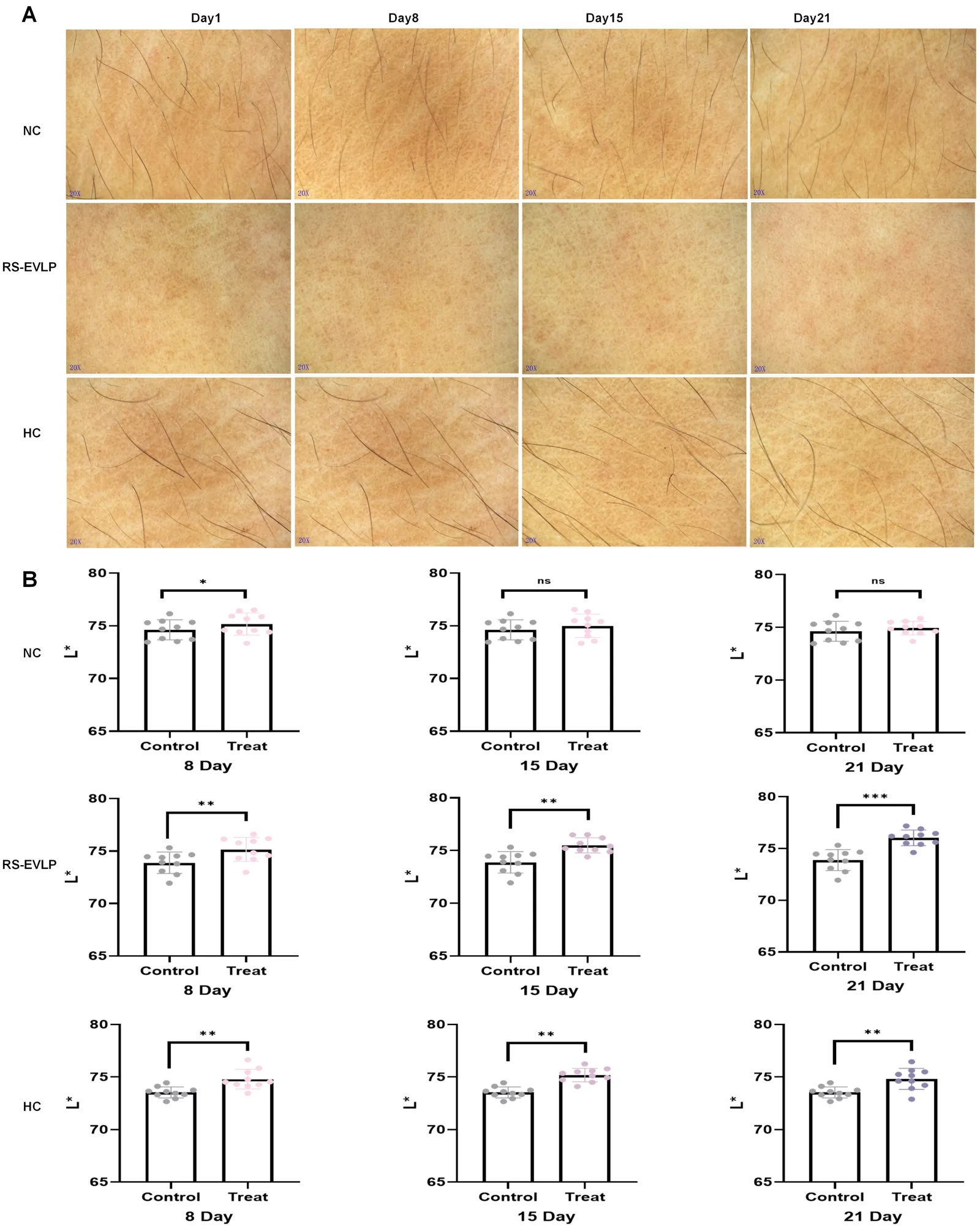
Clinical Assessment of the Efficacy of RS-EVLP on Human Skin Whitening. (A) Dermatoscopic images of representative areas of subjects’ skin on days 8, 15, and 21 after RS-EVLP application; (B) Skin luminance (L^*^) values over time in RS-EVLP- and HC-treated groups [baseline (day 0) and on days 8, 15, and 21] *vs*. the control group. ^*^P < 0.05; ^**^P < 0.01; ^***^P < 0.001. Data are expressed as the mean ± SEM from *n* = 10 biological replicates (independent volunteers), with each volunteer measured in triplicate at each time point (technical replicates, *n* = 3). Statistical significance was determined by repeated-measures two-way ANOVA followed by Bonferroni’s post-hoc test for multiple comparisons between groups at each time point. ^*^P < 0.05; ^**^P < 0.01; ^***^P < 0.001 *vs*. control group. ANOVA: Analysis of variance; HC: hydroquinone cream; NC: negative control; RS-EVLP: rose-derived extracellular vesicle-like particles; SEM: standard error of the mean.

## DISCUSSION

*R*. *rugosa*, a botanical treasure rich in bioactive components, such as aromatic alcohols, terpenoids, flavonoids, anthocyanins, and phenolic acids, has demonstrated a wide range of potential applications in dermatology due to antioxidant and anti-inflammatory properties^[47,48]^. The high vitamin C content, as well as levels of ellagic acid, anthocyanins, gallic acid, and quercetin, provide a solid scientific basis for using roses for the development of skincare products^[49-58]^. However, the whitening efficacy of RS-EVLP has not been extensively studied. In this study, we systematically elucidated the antimelanogenic properties of RS-EVLP and established potential for use in novel natural cosmeceuticals. RS-EVLP are bilayered nanoparticles measuring between 50 and 150 nm with physicochemical properties similar to those of synthetic liposomes; however, the natural botanical constituents endow RS-EVLP with multifunctional skin benefits. Mass spectrometry showed that RS-EVLP retained the major active components of the parent plant (roses). It is worth noting that the current use of botanical active ingredients is often limited by poor transdermal absorption. For instance, traditional rose extracts (e.g., ellagic acid and vitamin C) struggle to penetrate deeply into the epidermis due to poor water solubility and stability^[59,60]^. In this study, the bilayer nanostructure (50-150 nm) and natural lipid composition of the RS-EVLP enabled efficient penetration into the stratum corneum and delivery of active substances to the basal layer (as confirmed by transdermal experiments in nude mice), significantly enhancing efficacy^[61]^.

Compared with findings from other studies involving plant-derived extracellular vesicles, our melanogenesis results demonstrate both similarities and unique advantages. Although ginseng-derived extracellular vesicles have been shown to exhibit anti-melanogenic effects through MITF downregulation, their transdermal delivery efficiency has not been systematically evaluated in animal models. In contrast, our study not only confirmed the inhibition of the MITF-TYR signaling axis but also demonstrated efficient transdermal penetration in nude mice and confirmed whitening efficacy in a human clinical trial, providing a more comprehensive translational basis.

Following a review of the literature, we selected 47 active ingredients of RS-EVLP for network pharmacology analysis. Protein-protein interaction network analysis revealed that RS-EVLP could regulate TYR production. GO and KEGG pathway analyses further indicated that RS-EVLP were related to the melanogenesis pathway. Based on this, we consulted the KEGG website to identify the mechanistic pathways related to melanogenesis, which reported MITF, TYR, TRP-1, and TRP-2 as key targets. Using the pharmacological map of the intersection network between the constituent targets of RS-EVLP and pigmentation disease targets, we screened six core active ingredients corresponding to TYR: quercetin, lignocerotoxin, apigenin, kaempferol, ellagic acid, and proanthocyanidins. Due to limitations in the protein databases, MITF and TRP-1 were ultimately selected as pair acceptors. These six core active ingredients were then used as ligands for molecular docking to elucidate the potential molecular mechanism of the anti-pigmentation properties of RS-EVLP. Based on docking of target proteins and receptor components, we selected four combinations with the strongest binding energy (procyanidins/luteolin-MITF/TRP-1) for further molecular dynamics simulations and SPR analyses. The results showed that both RS-EVLP components bound stably to MITF/TRP-1, and that the binding of procyanidins to MITF/TRP-1 proteins was more stable (RMSD < 0.2 nm). The abundance of procyanidins was also higher in the RS-EVLP mass spectra. These findings reveal a molecular mechanism of RS-EVLP to inhibit melanin synthesis by targeting the MITF-TYR signaling axis through flavonoid components. Next, these conjectures were verified by *in vivo* and *ex vivo* experiments.

Flow cytometry and confocal microscopy revealed that B16 cells efficiently internalized RS-EVLP in a dose-dependent manner at concentrations ranging from 4.6 to 23 μg/mL, with no observed cytotoxicity [Figure 3A and D]. In terms of melanogenesis inhibition, RS-EVLP exhibited a significant, concentration-dependent effect. At 23 μg/mL, the RS-EVLP reduced melanin content by 16% and TYR activity by 26%. To investigate the molecular mechanism, we established an α-MSH induction model (validated by a 2.47-fold increase in MITF expression, *P* < 0.01) and confirmed that the RS-EVLP inhibited melanogenesis by regulating the downstream targets of the α-MSH-MC1R pathway. At a treatment concentration of 23 μg/mL, RS-EVLP significantly reduced protein levels of MITF, TYR, TRP-1, and TRP-2 by 41%, 40%, 44%, and 47%, respectively [Figure 3G-J]. This effect was directly associated with reduced melanin synthesis. Although MC1R upstream regulation was not directly detected, synchronized inhibition of the MITF-TYR axis suggests that RS-EVLP may act by blocking key nodes in the α-MSH signaling pathway.

Given that the skin layer structure (epidermis and dermis) of wild AB-type zebrafish is highly similar to that of humans and that melanogenesis mechanisms depend on TYR catalyzing the oxidation of tyrosine to dopaquinone, we chose wild AB-type zebrafish as an *in vivo* model. The results confirmed that the RS-EVLP significantly reduced the melanin content of zebrafish embryos. *In vitro* microscopic observation and ImageJ software analysis showed that the area of melanin spots was reduced by 24% ± 17.5% (*P* < 0.0103). Quantitative analysis determined the melanin content by NaOH cleavage and revealed that the melanin content of zebrafish litters in the RS-EVLP-treated group (0.46 mg/mL, 72 h) was reduced by 10.7% ± 2.8% (*P* < 0.01).

In addition, most plant extract studies are limited to cellular experiments and *in vivo* modeling, with no clinical data available. For instance, although strawberry-derived exosome-like nanoparticles have been shown to prevent oxidative stress in human mesenchymal cells, this has not been validated *in vivo* or in clinical trials. This study systematically verified the transdermal effect and whitening efficacy of RS-EVLP in an animal model and human clinical trial (ΔL^*^ +2.15, *P* < 0.0001), providing a direct basis for use in cosmeceuticals. Specifically, in the human clinical trial, topical application of a 5% RS-EVLP formulation increased the skin fairness of the volunteers, and the L-value of the volunteers in the RS-EVLP group continued to increase from day 8 (ΔL^*^ = 1.27, *P* = 0.0004) to day 21 (ΔL^*^ = 2.15, *P* < 0.0001) without exhibiting biotoxicity.

Although the present study revealed the remarkable efficacy of RS-EVLP for skin whitening, there are still some limitations. First, although network pharmacology and molecular docking predicted multiple active components and targets of RS-EVLP, the specific bioactive ingredients responsible for the anti-melanogenic effect and their direct modulation of cell signaling pathways have not been fully elucidated. Further systematic identification and validation are required. Second, the effect of RS-EVLP on gene expression upstream of the α-MSH-MC1R signaling pathway, including key regulators such as CREB and MITF, and their upstream kinases, was not experimentally verified. Third, the zebrafish *in vivo* experiment showed a relatively high variability (24% ± 17.5% reduction in melanin spot area), and the sample size in the clinical trial was small (*n* = 10). Fourth, the long-term safety and efficacy of RS-EVLP for skin whitening remain to be evaluated in larger-scale and longer-duration clinical studies. Fifth, the exact *in vivo* biodistribution, metabolism, and excretion profile of RS-EVLP are yet to be characterized. Addressing these limitations in future studies will further strengthen the translational potential of RS-EVLP as a natural whitening agent. Sixth, the B16 cells used in this study were not authenticated by short tandem repeat (STR) profiling, and routine mycoplasma testing was not performed. Although no contamination was observed during the experiments, the absence of these quality controls represents a limitation that should be addressed in future studies to ensure reproducibility and reliability. Addressing these limitations in future studies will further strengthen the translational potential of RS-EVLP as a natural whitening agent.

In summary, RS-EVLP are natural, non-toxic nanoparticles derived from plants that exhibited significant skin-whitening efficacy as a new generation of natural skin-whitening agents by reducing melanogenesis and enhancing transdermal delivery through the targetable MITF-TYR axis. RS-EVLP do not rely on chemical synthesis, in line with the concept of green chemistry and sustainable development. Future studies will further explore the direct modulation of the α-MSH-MC1R signaling pathway by RS-EVLP, with particular emphasis on measuring key upstream steps including cAMP production, PKA/CREB activation, and MC1R expression. Additionally, the involvement of other melanogenesis-related pathways (e.g., ERK, p38, Wnt/β-catenin) will be investigated to elucidate the comprehensive mechanism of RS-EVLP. Long-term clinical trials will also be conducted to evaluate the whitening efficacy and safety of RS-EVLP among individuals with different skin types.

## DECLARATIONS

### Availability of data and materials

All relevant data are included within the paper. The data that support the findings of this study are available from the corresponding author upon reasonable request.

### AI and AI-assisted tools statement

During the preparation of this manuscript, the AI tools DeepL Write (DeepL Write Next-gen) and DeepSeek (DeepSeek R1) were used solely for grammar and spelling editing. These tools did not influence the study design, data collection, analysis, interpretation, or the scientific content of the work. All authors take full responsibility for the accuracy, integrity, and final content of the manuscript.

### Copyright

© The Author(s) 2026.
