# Supplementary material for: Rose-derived extracellular vesicle-like particles enable anti-melanogenesis by suppressing the key melanogenic pathway in skin cells
Source: Extracell Vesicles Circ Nucl Acids. 2026 Sep 4;7(3):1388–410. doi: 10.20517/evcna.2026.55 (PMC13673352; doi:10.20517/evcna.2026.55)
Supplement: Supplementary file 1 [file evcna-7-3-1388-SupplementaryMaterials.pdf]

Supplementary Materials

Rose-derived extracellular vesicle-like particles enable anti-melanogenesis by suppressing the key melanogenic pathway in skin cells

Sirui Zhou[1,2,3], Huijing Li[3], Ying Yang[3], Weiying Zeng[3], Dongxiao Li[1,3], Tianxin Qiu[1,3], Weiguo Lu[2], Kewei Zhao[1,3]

[1]The Third Affiliated Hospital of Guangzhou University of Chinese Medicine, Guangdong Engineering Research Center of Chinese Herbal Vesicles, Guangzhou 510378, Guangdong, China.
[2]The First Affiliated Hospital of Guangzhou University of Chinese Medicine, Guangzhou 510405, Guangdong, China.
[3]The Third Clinical Medical College, Guangzhou University of Chinese Medicine, Guangzhou 510378, Guangdong, China.

Correspondence to: Dr. Kewei Zhao, The Third Clinical Medical College, Guangzhou University of Chinese Medicine, Guangzhou 510378, Guangdong, China. E-mail: zkw2011@gzucm.edu.cn; Dr. Weiguo Lu, The First Affiliated Hospital of Guangzhou University of Chinese Medicine, Guangzhou 510405, Guangdong, China. E-mail: 3897988@qq.com

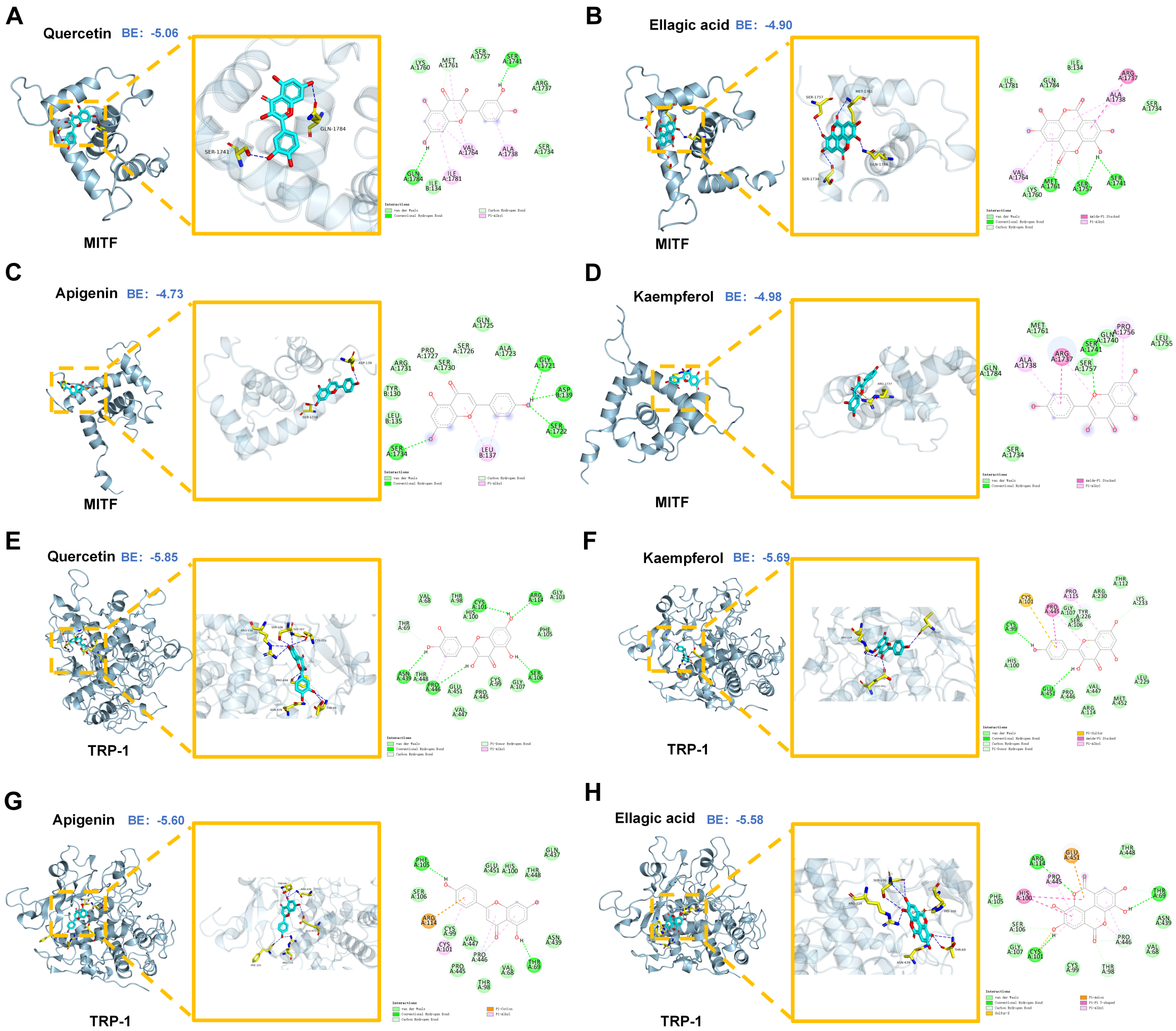

Supplementary Figure 1. Molecular docking binding modes of four flavonoid active components of RS-EVLP with target proteins. (A-H): Three-dimensional molecular docking interaction diagrams of key active components [(A and E) quercetin; (B and F) ellagic acid; (C and G) apigenin; and (D and H) kaempferol] with the functional binding pockets of the MITF and TRP-1 proteins (energy-optimized conformations shown).
